# Gastric Hyperæsthesia and Acidity

**Published:** 1892-02-20

**Authors:** 


### The Practitioner's Mirror and Retrospect.

[*The Editor will be glad to receive offers of co-operation and contributions from members of the profession. All letters should be addressed to* The Editor, The Lodge, Porchester Square, London, W.]

### MEDICINE.

### GASTRIC HYPERÆSTHESIA AND ACIDITY.

The connection between gastric disease and disorders of the nervous system formed the subject of a contribution by M. Cuffer, in a recent number of the *Revue de Médicine*, in which he endeavoured to prove that reflex action cannot explain the persistency with which nervous disorders appear in connection with diseases of the stomach. In chronic gastric cases, notably in those of cancer, he has observed the presence of these disturbances, and he considers it possible that they may depend upon an ascending inflammation of the pneumo-gastric nerve, extending to the bulb, and on this supposition he explains the bulbar symptoms which he has found present in his cases during life, and which post-mortem examination has enabled him to verify. It will be difficult for practitioners to acquiesce in these views, but it is well known that some severe forms of gastric pain are dependent on disease of the nervous system.

The effect the excessive formation of acid in causing great pain in the stomach, appears to be more in accordance with general observation. In a paper on hyperæsthesia of the stomach, more especially of its mucous membrane, Rosenheim* quotes eight cases in young girls of between sixteen and twenty-two years of age suffering from chlorosis and anæmia.

The connection between blood-disease and stomach-affections is proved beyond a doubt. In chlorosis and anæmia of a slight degree, the affection of the blood is primary, that of the stomach secondary. First there is often anorexia in varying degrees, as a sign of the disturbances of sensibility in the nerves that give the feeling of hunger. The normal sensibility of the stomach is slight, while pathological disturbances vary to an extraordinary degree in the symptoms. The history given in these cases was that there appeared—usually slowly, but in a few instances somewhat rapidly— a condition of great irritability of the gastric mucous membrane, showing itself in the mildest form by a feeling of weight after food, and going on in later stages to such intolerance that not only nausea and sickness occurred, but likewise almost uncontrollable vomiting, which lasted until all the food taken was rejected. The epigastrium was tender, but there was no apparent derangement of the motor or secretory gastric functions; and though disturbance of appetite was several times present, it was never excessive. In the milder cases there was a feeling of pressure after eating, and in the more severe forms there was intractable vomiting. There were also symptoms of hyperæsthesia in the adjacent parts, probably emanating from the sympathetic system. In these conditions, argentum nit. (0·2 : 100·0) was given, half a table spoonful in a wineglass of water three times a day, with excellent results. The diet was strictly controlled, and consisted of milk in small quantities, with lime water; later on, tea or coffee with milk, and weak veal broth; in special cases yolk of egg, with sugar and a little brandy, grated meat cakes and biscuit. When intolerance of the stomach subsided, and more solid food could be given, bitter tonics were administered if the appetite was poor, and then only arsenious acid with iron was prescribed.

He did not observe nervous symptoms, ventricular ulcer, or gastric catarrh. Moller and Leube were the first to point to the occurrences of gastric hyperæsthesia, especially in anæmic women. But Rosenheim found various forms of neuralgia in many cases. Cardialgia is, in its neuralgic character, separated from hyperæsthesia, as the latter does not occur spontaneously with lightning-like quickness, but is always caused by the ingesta of the stomach, or by external pressure. Rosenheim adduces two cases, in which, in addition to hyperæsthetic disturbances, there were symptoms of hyper-acidity (acid eructations and vomiting, and a gnawing pain). Lactic acid and hydrochloric acid were plainly observable in both cases. In the ten cases observed by Rosenheim there was independent gastric neurosis, but an anatomical basis is very probable. There may have been erosions and epithelial waste of the mucous membrane, which is particularly vulnerable in anæmic subjects. This would give the best explanation of the existing hyperæsthesia, as well as of the success of the treatment with argentum.

* *Wien. Med. Woch.*, No. 6, 1891; *Lond. Med. Rec.*, Mar. 20th, 1891; *Berl. Klin. Woch.*, No. 33, 1890; *Pract.*, Dec., 1890.

In this connection the case of a man, aged 20, under Dr. J. C. Taylor * is of great interest. He came with a history of intense abdominal pain of a duration of two years and a half, unknown as to its cause and unrelieved by any remedy. The family history was very good, but with a tendency to gout on the father's side. Before the commencement of the present illness there was nothing particular in the history of the patient. Without any exciting cause he began to be troubled with pain in the epigastric and left hypochandriac regions. The pain from the beginning was always severe, with a tendency to be worse during the night, and to disappear or abate during the day. Sometimes he would be free for a week or more; but the symptoms always returned with their usual vehemence. Latterly, the intensity of the pain seemed to have been increasing. The locality appeared to have been always the same, and to be represented by the outer two-thirds of the left costal margin and downwards for about two fingers' breadth. If a paroxysm increased in violence, pain began to be felt in the lower dorsal region, and as it grew worse pain spread upwards in a line along both sides of the spinal column, and when at its worst the sensation was described "as if a red-hot needle was being run up and down the middle of the back." After trying various remedies, he decided to give bicarbonate of soda, and commenced with sixty grains; the effect was simply astonishing; in two or three minutes there was an eructation and total relief of pain. After this sixty grains of the antacid was given whenever the symptoms recurred. The result was always the same, viz., relief in from three to five minutes with an eructation, but the length of time between the doses varied from thirty minutes to four or five hours. For the first few days eleven or twelve doses in the twenty-four hours were necessary, and after a week eight or nine were the average.

The effects of drachm doses of the antacid were simply marvellous, yet any less dose had little or no effect. Other antacids in similar large doses were given, but none of them acted with the rapidity or gave the same relief as the bicarbonate of soda. On two occasions whilst taking the antacid the patient vomited a large quantity of watery fluid, sour smelling, each time the ejected matter being in much larger quantities than the fluid previously imbibed. The action of the sulphonal which had also been prescribed appeared not so much to give sleep as to act as an analgesic, and thus allow natural sleep, for if it was left off for a night, or diminished below ten grains, sleep was obtained, but was mere broken by attacks of pain, and more doses of the antacid had to be taken in the night, but not during the following day. The action of the carbolic acid was, no

* *Lancet*, Dec. 6th, 1890.

doubt, an analgesic and antiseptic, preventing the fermentation that was probably the cause of the abnormal quantity of acid.

The remarks of Dr. Germain Sée, * on the value of Cannabis Indica in gastric affections are worthy of careful note. Dr. Sée arrives at the following conclusions: 1. The most convenient form in which to employ the drug is the extract in doses of about three-quarters of a grain daily, divided into three portions. Above this dose the drug is apt to produce unpleasant effects. (The French extract is stronger than the English). 2. The drug was chiefly tried on the non-organic affections of the stomach. These were divided into two groups. The first included cases in which the gastric juice was altered in composition, especially if there was an excess of hydrochloric acid. The second group consisted only of cases of gastro-intestinal neuroses, in which there was no change in the digestive juices. 3. All these affections—dyspepsias and neuroses—were characterised by five sets of symptoms, occurring in various proportions: (*a*) Pain, local or radiating, arising spontaneously or after food. The variations in appetite belong to this group. (*b*) Atony of the stomach, with or without dilatation, is almost always present. Vomiting is more frequent in the neurotic cases. (*c*) Flatulence and eructation occur in most cases; in the neuroses the gas consists chiefly of air which has been swallowed; gases formed by decomposition arise from lactic or acetic acid fermentation, and not from excess of hydrochloric acid. These gases are the cause of the painful symptom known as "heartburn." (*d*) The gastric digestion of flesh food and albuminoids is little affected when hydrochloric acid only is in excess, but it is deficient when too much lactic or acetic acid is present, and in abeyance when there is deficiency of hydrochloric acid. In the neurotic cases gastric digestion is normal. Constipation is the rule in most cases. (*e*) In this last group are placed the varied symptoms—giddiness, migraine, palpitation, agoraphobia, &c. 4. Cannabis indica gives relief from pain and increases the appetite in all cases, no matter on what causes the pain and loss of appetite may depend. If, however, too much hydrochloric acid be excreted, it is better to aid the action of the drug by large doses of bicarbonate of soda, given about four hours after food. Cannabis indica has no beneficial action on the atonic state of the stomach, but it relieves vomiting and cramp of the stomach.

* *Deutsch. Med. Woch.*, Aug., 1891; *Lancet*, Sept. 20th, 1891.

